# MicroRNAs in IgA nephropathy

**DOI:** 10.1080/0886022X.2021.1977320

**Published:** 2021-09-22

**Authors:** Xingchen Yao, Yaling Zhai, Huanping An, Jingge Gao, Yazhuo Chen, Wenhui Zhang, Zhanzheng Zhao

**Affiliations:** a Department of Nephrology, the First Affiliated Hospital of Zhengzhou University, Zhengzhou, China; b The Renal Research Institution, Zhengzhou University, Zhengzhou, China; c Medicine Experiment Center, Hanzhong Vocational and Technical College, Hanzhong, China

**Keywords:** MicroRNA, IgA nephropathy, pathogenesis, diagnosis, prognosis

## Abstract

IgA nephropathy (IgAN) is the most common primary glomerulonephritis worldwide. It is considered that the pathogenesis of IgAN involves the ‘multiple hit theory’ and the immune-inflammatory mechanism; however, these theories have certain limitations. The gold standard for diagnosing IgAN is still renal biopsy. Although renal biopsy is accurate, it is traumatic and is associated with some risks and limitations, so there is a need for non-invasive diagnostic methods. According to recent studies, microRNAs (miRNAs) play important roles in the occurrence and development of IgAN; thus, they provide the possibility of the noninvasive diagnosis of IgAN and also have some value in predicting prognosis. This review summarizes the current research status of miRNAs in the occurrence, development, diagnosis, and prognosis of IgAN. We also highlight some interesting and challenging points that require further study.

## 1. Introduction

IgA nephropathy (IgAN) is the most common primary glomerulonephritis in the world. Approximately 20–40% of patients with IgAN develop the end-stage renal disease (ESRD) within 10–20 years [1,2]; therefore, IgAN is one of the main causes of ESRD [3–7].

The research of microRNAs (miRNAs) in IgAN began with the study of miRNAs in urine sediment of patients with IgAN by Wang et al. in 2010 [8]. That study described the relationship of the urinary sediment miR-200 family, miR-205, and miR-192 in IgAN with the severity of IgAN renal injury and IgAN prognosis; it even speculated the mechanisms by which these three miRNAs affect renal fibrosis and provided new ideas for the study of miRNAs in IgAN. Subsequently, in 2012, Serino et al. [9] reported that the deregulation of miR-148b was a key in the pathogenesis of IgAN, which could result in galactose­deficient IgA1 (gd-IgA1) in IgAN. Since then, studies of miRNAs have mainly focused on the mechanisms by which miRNAs influence the pathogenesis of IgAN, the association between miRNAs and the severity of IgAN, and the relationship between miRNAs and the prognosis of IgAN. In recent years, the potential value of miRNAs as biomarkers in IgAN has gradually developed, especially for miRNAs in urine sediment. MiRNAs in urinary sediment have been explored as noninvasive biomarkers of IgAN. In this article, we provide a detailed review of the effects of miRNAs on the pathogenesis and development of IgAN, their relationship with the severity of renal damage in IgAN, as well as their potential in diagnosis and predicting prognosis of IgAN.

## 2. MiRNAs and the pathogenesis of IgAN

### 2.1. MiRNAs and gd-IgA1

Gd-IgA1 plays an important role in the pathogenesis of IgAN. Most studies about miRNAs and the pathogenesis of IgAN are related to gd-IgA1.

It has been demonstrated that C1GALT1 could regulate the production of gd-IgA1 in IgAN [10,11]. Using high-throughput analysis, Serino et al. [9] found that miR-148b was upregulated in peripheral blood mononuclear cells (PBMCs) of IgAN. Based on an in silico analysis, they found that the gene C1GALT1 was a potential target of miR-148b. The increase in miR-148b level could lead to the reduction of C1GALT1, both at the RNA and protein expression levels. At the same time, they found that the 1365 G/A polymorphism (rs1047763) in C1GALT1 3′-UTR affected the miR-148b binding site in this gene, and the biocomputational analysis showed that 1365 G allele enhanced miR-148b binding. Moreover, the upregulated expression of miR-148b was specific to IgAN compared with other forms of nephropathy. Serino et al. further validated the relationship of miR-148b and gd-IgA1 in IgAN; the upregulation of miR-148b positively correlated with the serum level of gd-IgA1. After transfecting human B lymphoma DAKIKI cells with miR-148b mimics and inhibitors, the authors analyzed the gd-IgA1 levels by HAA lectin binding analysis and confirmed the direct causal relationship between miR-148b and gd-IgA1. These data further confirmed the pivotal role of miR­148b in the regulation of IgA1 O-glycosylation. In summary, this in-depth study validated the relationship of mir-148b and gd-IgA1, found the direct target of mir-148b, and explored how mir-148b acted on C1GALT1. However, C1GALT1 is not the only target of miR-148b. Serino et al. [12] revealed that another miRNA, let-7b, participated in the generation of gd-IgA1. They found that let-7b was significantly upregulated in PBMCs of IgAN patients, and the upregulation of let-7b was specific to IgAN compared with other forms of nephropathy. The bioinformatic analysis revealed that GALNT2, the key enzyme for O-glycosylation of IgA1, was the potential target of let-7b. qRT-PCR and western blot analysis revealed that GALNT2 was significantly downregulated in IgAN. Both at RNA and protein levels, higher let-7b levels were associated with lower levels of GALNT2. The study also demonstrated that let-7b was upregulated in B-lymphocytes in IgAN patients. However, the study was limited to participants of Caucasian ancestry.

Cosmc is a unique molecular in mammalian galactosyltransferase [13]. According to Hu et al. [14], Cosmc is reduced in IgAN; Cosmc is associated with gd-IgA1 In IgAN, the level of Cosmc in B cells transfected with miR-374b ASO increased significantly, while the level of gd-IgA1 decreased. These results indicate that miR-374b acts on Cosmc, leading to gd-IgA1 in IgAN. Although there was a validation cohort in that study, the number of patients in the validation cohort was small: 25 in the IgAN group and 10 in the normal control group. Therefore, the reliability of the experimental results needs to be further verified. Lichuan Yang [15] found miR‑155 deficiency in the PBMCs of patients with IgAN. The analysis showed significantly reduced expression of Foxp3 and Cosmc in PBMCs of IgAN patients. ELISA analysis revealed that the serum gd-IgA1 level in IgAN patients was higher than that in normal controls. In addition, significant correlations were found between the miR-155 level and Foxp3, Cosmc, and gd-IgA1 levels. Taken together, miR-155 may lead to the formation of gd-IgA1 by inhibiting the expression of Cosmc. However, miRNA analysis was performed on PBMCs rather than on isolated lymphocyte subgroups because of the insufficient amount of RNA isolated from lymphocyte subgroups; therefore, further *in vitro* experiments are required to verify this hypothesis. In addition, Chunmei L speculated that overexpression of miR-320 decreased Cosmc expression in B cells [16].

HMGB2 participates in four IgAN-related pathways, namely, the inflammatory response, defense response to bacteria, diversification of immune receptors, and cell surface receptor signaling pathways [17,18]. Zhai et al. used three gene expression profile datasets (GSE14795, GSE73953, and GSE25590), and the differentially expressed genes (DEGs) and miRNA network associated with IgAN constructed by Cytoscape, to screen out the associations of HMGB2 and hsa‐miR‐590‐3p with IgAN [19]. The dual‐luciferase reporter system indicated that hsa‐miR‐590‐3p bound to the 3′-UTR of HMGB2 and inhibited its expression. They found that hsa‐miR‐590‐3p increased, while HMGB2 decreased in PBMCs of IgAN patients, indicating a significant negative correlation between the expression of HMGB2 and hsa-miR-590-3p. However, the mechanism by which HMGB2 affects the production of gd-IgA1 is still unclear and needs further research.

### 2.2. MiRNAs and immunoregulatory disorder

About 35–50% of patients with IgAN have elevated serum IgA levels, which are closely related to B and T cells. It is believed that p-IgA in IgAN is produced by polyclonal B cells, while IgA secreted by B cells is regulated by T cells. Dysfunctional immune regulation of T cells can cause uncontrolled and excessive production of IgA by B cells [20]. Previous studies have suggested the relationship between miRNAs and immunoregulatory disorders. According to Xu et al. [21], miR-21-5p drives the polarization of T-helper cells in IgAN, clearly showing the relationship between miRNAs and immune regulation imbalance. First, through miRNA sequencing, miR-21 was screened out as it had the highest expression level in PBMCs. Then, computer analysis predicted that SPRY1, SPRY2, and FASLG are the target genes of miR-21 in IgAN. In another independent study population (study population 2), CD3+ T lymphocytes were identified as the lymphocyte subset responsible for the upregulation of miR-21 expression in IgAN; these results were verified in another independent study population (study population 3). Compared with the healthy control group, patients with IgAN had a significantly higher expression level of miR-21 in CD3+ T cells and significantly lower expression levels of SPRY1, SPRY2, and FASLG. The proportion of Th17 cells was elevated in the IgAN group, and it was negatively correlated with SPRY1 expression; however, there was a positive correlation between the proportion of Th17 cells and IgA1 level. Overall, these results showed that upregulation of miR-21 expression in T lymphocytes suppressed the expression of SPRY1, SPRY2, and FASLG, and it induced Th17 polarization in patients with IgAN.

MiR-155 is a multifunctional immunomodulatory miRNA [22,23]; it is involved in T regulatory (Treg) cell adaptability [24] and Th1/Th2 balance [25,26]. Yang et al. [15] confirmed that the expression level of miR-155 in PBMCs in IgAN was significantly reduced and caused the drift of T lymphocyte subsets (Th2 and Th17 increased, whereas Th1 and Treg decreased). The levels of Th2 cytokine (IL-5) and Th17 cytokine (IL-17) were significantly higher, while those of Th1 cytokine (INF-γ) and Treg cytokine (IL-10) were lower in patients with IgAN. The authors speculated that such ‘drift’ would cause the suppression of the *Cosmc* gene and the production of gd-IgA1, eventually leading to the deposition of IgA in the kidneys. Jin et al. [27] proved that miR-133a and miR-133b inhibited the Treg differentiation in IgAN by targeting FOXP3. According to other studies, miR-146a [28] may regulate Th2 cells, and miR-320 [16] and miR-374b [14] promote B cell proliferation by inhibiting PTEN expression.

In general, the available studies on the relationship between miRNAs and immune regulation were not able to directly measure the content of miRNAs from differentiated lymphocyte subsets because of the low content of miRNA in lymphocyte subsets. Perhaps expanding the sample size or testing *in vitro* cell culture can solve this problem.

### 2.3. Other potential aspects of pathogenesis related to miRNAs

There are some miRNAs whose mechanism in IgAN has not yet been demonstrated. However, it has been shown that these miRNAs are associated with the pathogenesis of other kidney diseases, or there are clues that they may play a role in the pathogenesis of IgAN. Therefore, these miRNAs also have a great research value, providing a direction for future research. For example, miR­106b­5p and miR­17­5p regulate cell­cycle progression and apoptosis [29,30]. MiR-17-5p also participates in the regulation of endocytic transport by targeting the GTPase activator protein TBC1D2 [31]. A high­throughput sequencing analysis [32] showed increased expression levels of miR­106b­5p and miR­17­5p in IgAN, suggesting that miR­106b­5p and miR­17­5p might participate in proliferation and endocytic trafficking in mesangial cells. In addition, there has been new evidence that miR­133a, miR­133b, and miR­185 are involved in the deposition of IgA1 in the mesangium [32]. These results provide some important clues for future studies.

**Figure 1.**
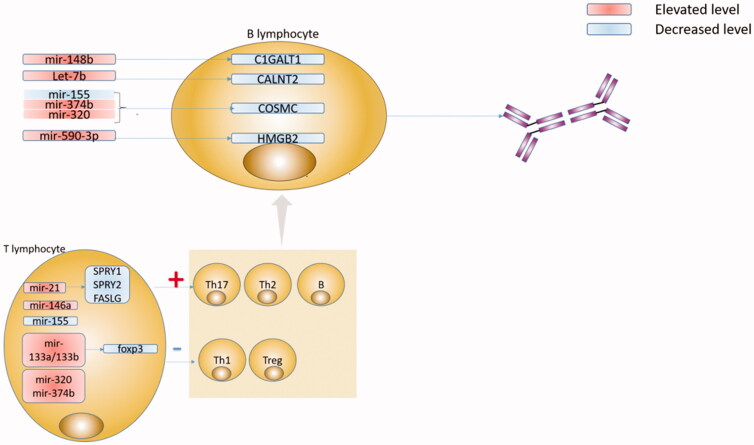
Specific microRNAs (miRNA), their putative targets, and pathophysiological effects in IgA nephropathy (IgAN). A number of steps in the pathogenesis of IgAN are regulated by miRNA: upregulated miR-148b inhibits C1GALT1, upregulated let-7b inhibits CALNT2, upregulated miR-374b, miR-320, and downregulated miR-155 inhibits COSMC in B lymphocyte. These enzymes had been demonstrated important roles in the molecular basis for the aberrant IgA1 glycosylation in IgAN. Meanwhile, upregulated miR-590-3p inhibits HMGB2 in B lymphocytes, and the mechanism HMGB2 affects the production of gd-IgA1 remains unclear and needs further research. The immunoregulatory disorder is another important mechanism in IgAN. In T lymphocyte, upregulated miR-21 inhibits SPRY1, SPRY2 and FASLG, upregulated miR-133a/133b inhibits foxp3, and upregulated miR-146a, miR-320, miR-374b, and downregulated miR-155 induces Th17 polarization and influence the fitness of Treg cells and the Th1/Th2 balance.

In conclusion, miRNAs are likely involved in several pathways of IgAN pathogenesis, and multiple miRNAs are involved in one pathogenesis pathway as well. C1GalT1, Cosmc, FOXP3, and PTEN are the main targets of miRNAs, which are regulated by multiple miRNAs and participate in multiple pathways. Overall, miRNAs and their targets comprise a complex network involved in the pathogenesis of IgAN. In order to better understand the pathogenesis of IgAN, it is necessary to find a ‘central’ miRNA and its target, which is highly specific and associated with multiple classic pathways of pathogenesis. We summarized IgA pathogenesis related to miRNAs in Figure 1.

## 3. MiRNAs and the progression of IgAN

### 3.1. MiRNAs and renal interstitial fibrosis

The miR-29 family plays an important role in the progression of IgAN. Previous studies in rats or mice have shown that downregulation of miR-29 family members could exacerbate renal interstitial fibrosis, while their overexpression can attenuate fibrosis [33,34]. It has been found that the expression of miR-29 in the renal cortex of 5/6 nephrectomized rats treated with L-Mim was increased, thereby significantly reducing the interstitial fibrosis of the residual kidney [34]. MiR-29 also reduced interstitial fibrosis in cultured human renal epithelial HK2 cells by activating HIF-a. TPM1 and COL2A1 are predicted to be miR-29c targets. These conclusions have been initially verified in IgAN. Real-time PCR analysis showed that the expression level of miR-29c was significantly reduced in IgAN patients with renal interstitial fibrosis. Immunohistochemical analysis showed that the degree of substantial interstitial fibrosis in patients with IgAN was directly proportional to the levels of TPM1 and COL2A1. However, the sample size of this study was limited given that only 10 IgAN patients were included: five without interstitial fibrosis and five with moderate to severe interstitial fibrosis. Therefore, these results need to be further verified. MiR-21-5p, miR-199a-5p, and miR-214-3p are recognized as ‘fiber miRNAs’ [35]. These miRNAs are mainly upregulated through the TGF-β signaling pathway during fibrosis [36,37]. Hennino et al. [37] explored the relationship of miR-21-5p, miR-199a-5p, and miR-214-3p with the pathological changes of IgAN. In patients with glomerular and interstitial fibrosis, the expression levels of miR-21-5p, miR-199a-5p, and miR-214-3p increased significantly. Compared with patients with mild fibrosis, patients with moderate or severe fibrosis showed increased expression levels of miR-21-5p and miR-214-3p in the kidney tissue. Renal expression of miR-199a-5p was significantly increased only in patients with severe fibrosis, but not in patients with mild and moderate fibrosis. Based on *in situ* hybridization in renal tissue, it was further confirmed that miR-21-5p was expressed in the area of interstitial fibrosis, and the increase in miR-21-5p expression was related to the severity of interstitial fibrosis. However, the study lacked a validation cohort and disease controls; moreover, it did not profoundly explore how mir-21-5p affects renal interstitial fibrosis. Another study [33] revealed that miR-382 led to tubulointerstitial fibrosis in mice with unilateral ureteral obstruction, and the target of miR-382 was HSPD1. HSPD1 is a key protein that maintains mitochondrial integrity and cell viability, and miR-382 protects cells from oxidative stress by acting on HSPD1 [38,39]. IgAN with renal interstitial fibrosis was associated with upregulated miR-382/3-NT and downregulated HSPD1/Trx. MiR-192 was also upregulated in fibrotic kidney disease and led to renal fibrosis via the TGF-β/Smad signaling pathway [40]. Some scholars believe that the inhibition of renal miR-192 could ameliorate renal fibrosis [41]. However, Fan et al. [42] showed that lower intrarenal and serum miR-192 levels were related to higher interstitial fibrosis and renal tubular atrophy. The expression levels of renal E-cadherin and TGF-β1 in IgAN negatively correlate with serum miR-192.

Some other miRNAs have also been implicated in renal interstitial fibrosis in IgAN. In IgAN, through epithelial-to-mesenchymal transition (EMT), activated renal tubular epithelial cells can turn into activated fibroblasts, thereby favoring renal fibrosis [43]. Previous studies have shown that many miRNA species (such as miR-141 and miR-200b, miR-205 and miR-192) are associated with EMT. Wang et al. [8] proposed that in IgAN, the miRNA 200 family can inhibit EMT by directly inhibiting the E-cadherin repressors ZEB1 and ZEB2, but these results should be further verified. In the process of renal fibrosis, TGF-β1 strictly regulates the expression levels of miR-21, the miR-29 family, miR-93, miR-377, miR-216a, the miR-200 family, and miR-192 through SMAD3-dependent mechanisms [44]. Wang et al. [45] speculated that miR-21, miR-29, and miR-93 may affect renal fibrosis in IgAN.

**Figure 2.**
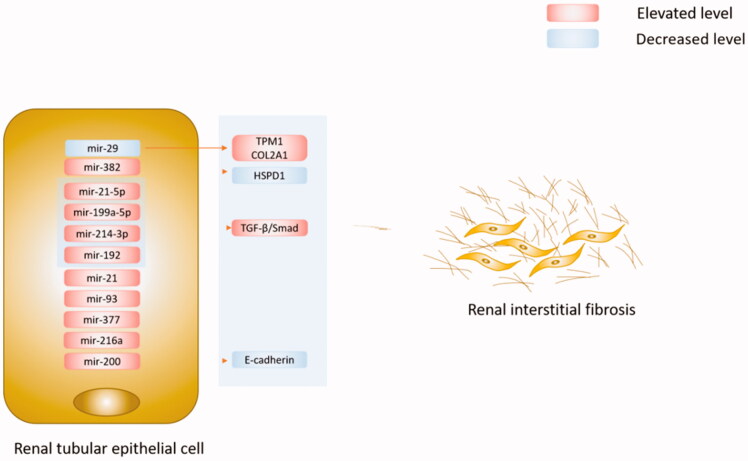
Specific miRNAs played important roles in the renal interstitial fibrosis of IgAN. Decreased miR-29 led to the aggregation of renal interstitial fibrosis. TPM1 and COL2A1 were predicted targets of miR-29c. Elevated miR-382 contributed to the progression of renal tubulointerstitial fibrosis, and the target of miR-382 was HSPD1. MiR-21-5p, miR-199a-5p, and miR-214-3p are three well-established ‘fibro miRNAs’. They and miR-192 contributed to renal interstitial fibrosis via the TGF-β/Smad signaling pathway. MiRNA 200 family can suppress EMT by directly inhibiting E-cadherin. In addition, miR-21, miR-93, miR-377, and miR-216a all were key microRNAs in renal interstitial fibrosis.

In general, the previous studies focusing on miRNAs and IgA nephrotic interstitial fibrosis were mainly based on clinical correlation analysis. However, renal interstitial fibrosis is not specific to IgAN; it is a necessary stage in many chronic kidney diseases. Therefore, future studies should pay attention to the mechanisms of interstitial fibrosis in IgAN regulated by specific miRNAs. We summerized the role of miRNAs in the renal interstitial fibrosis of IgAN in Figure 2.

### 3.2. MiRNAs and inflammation

**Figure 3.**
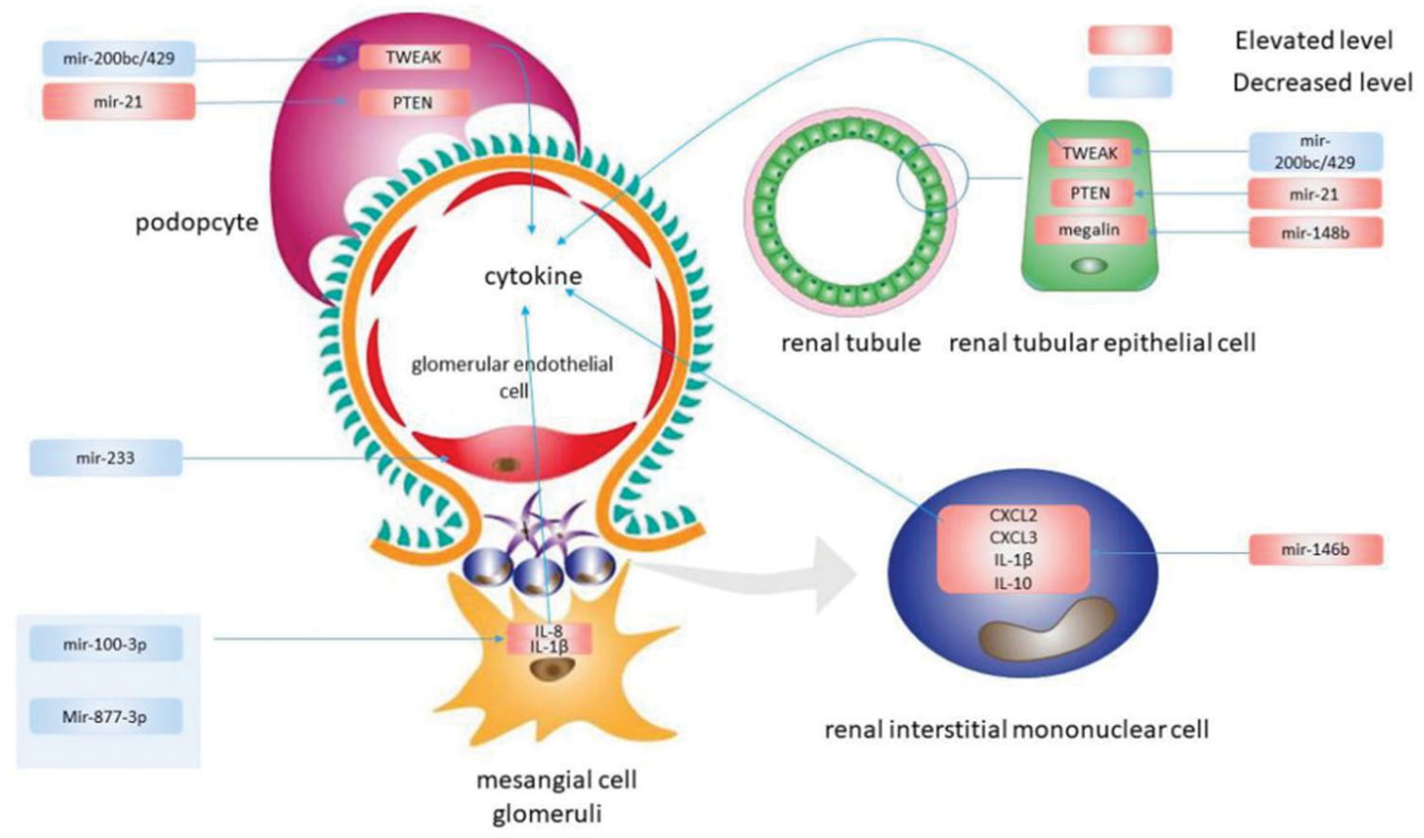
Inflammation is another important factor in the progress of IgAN. A variety of microRNAs acted on different cells to cause the release of inflammatory factors, thereby causing inflammation. In podocyte cell and renal tubular epithelial cell, decreased miR-200bc/429 targeted to TWEAK, leading to the release of inflammatory cytokines. In renal interstitial mononuclear cell, elevated miR-146b leaded to the release of CXCL2, CXCL3, IL-1β, IL-10. In mesangial cell, decreased miR-100-3p and miR-877-3p contributed to the release IL-8 and IL-1β. In addition, decreased miR-233 acted on glomerular endothelial cell, and leaded to the glomerular endothelial proliferation. And in podocyte cell and renal tubular epithelial cell, elevated miR-21 and miR-148b leaded to podocytes and tubular cells injure by targeting PTEN and megalin, respectively.

Inflammation plays an important role in IgAN and is closely related to disease progression and poor prognosis [46,47]. Gd-IgA1 and its specific antibody form circulating immune complexes (CIC), which are deposited in the glomerular mesangium and trigger renal injury. This further leads to the proliferation of mesangial cells and the release of proinflammatory and profibrotic mediators, which can directly lead to mesangial proliferation, podocyte injury, and extracellular matrix (ECM) generation, eventually resulting in ESRD. It has been shown that miRNAs can participate in the inflammatory response of IgAN by activating various inflammatory factors [48–53]. It has been shown [48] in chronic renal insufficiency models that the expression of miR-146a in the kidney and urine is significantly related to interstitial lesions and inflammatory cell infiltration. The expression of miR-146a also positively correlates with the expression of Cxcl2, Cxcl-3, IL-1b, and IL-10. The targets of miR-146a, IL-1 receptor-associated kinase 1 (IRAK1), and TNF receptor-associated factor 6 (TRAF6) are important components in TLRs and pro-inflammatory signaling pathways [50]. Although it has been shown that miR-146b is associated with IgAN [27,50], the mechanism by which miR-146a contributes to inflammation in IgAN needs further research. In another study, the miRNA expression in human renal mesangial cells (HRMCs) stimulated by serum IgA has been detected by bioinformatic approaches, filtering out miR-100-3P and miR-877-3P [53]. It has been shown that down-regulation of miR-100-3p expression in glomerular mesangial cells can stimulate serum IgA-induced overproduction of IL-8, while downregulation of miR-877-3p expression can lead to overproduction of IL-1β. Members of the miR-100 family play a very important role in the occurrence of tumors and inflammation [51], and miR-877 is related to the pro-inflammatory and immune regulation in the pathogenesis of Sjögren syndrome [52]. These results provide new clues for the pro-inflammatory mechanism of serum IgA in IgAN. In addition, another study found that miR-200bc/429 cluster could inhibit inflammation [54], namely, the expression of miR-200bc/429 cluster was downregulated in IgAN renal tissues, IgAN podocytes, and HK2 cells. In contrast, the overexpression of the miR-200bc/429 cluster can prevent the TWEAK-mediated NF-κB pathway in IgAN, thereby inhibiting the release of inflammatory cytokines, which provides a new strategy for the treatment of IgAN. We summerized the role of miRNAs in the inflammation of IgAN in Figure 3.

### 3.3. MiRNAs and other mechanisms of IgAN progression

In addition to the aforementioned mechanisms of interstitial fibrosis and inflammation, other mechanisms are gradually being discovered. It has been shown that diffuse glomerular endothelial cell hyperplasia is related to capillary hyperplasia, glomerular sclerosis, and severe proteinuria [55]. Glomerular endothelial cells cultured in a medium containing polymeric IgA have a reduced level of miR-223, and the level of miR-223 in glomerular tissues of patients with glomerular endothelial cell proliferation is also decreased [56]. MiR-233 downregulation promotes glomerular endothelial cell activation by upregulating importin alpha4 and alpha5 in IgA nephropathy [56]. It has also been found that miR-21 may be related to the damage of IgAN podocytes and renal tubular cells [57]. In particular, in the glomeruli and tubulointerstitial tissue of cases with IgAN, the levels of miR-21 located in podocytes and renal tubular cells are upregulated, and the target of miR-21 is PTEN. Inhibition of miR-21 can prevent the activation of the PTEN/Akt pathway in IgAN, thereby preventing the fibrotic activation of podocytes and renal tubular cells. These two experiments have been fully verified *in vivo* and *in vitro*. Recently, a study [58] showed that renal miR-148b is related to the downregulation of megalin in IgAN. Urine megalin has been identified as a potential biomarker of renal tubular damage in diabetic nephropathy and IgAN [59].

In conclusion, miRNAs are involved in IgAN through a complex network. A single miRNA can be involved in both pathogenesis and progression of IgAN, while different miRNAs are connected by the same targets. For example, in IgAN, the miR-200 family participates in renal interstitial fibrosis by ZEB1 and ZEB2, and in inflammation by TWEE/Fn14 [8]. MiR-21 is associated with Th17 proliferation and is involved in renal interstitial fibrosis as well as podocyte and renal tubular injuries [21,57]. MiR-155 acts on both Treg development by stimulating FOXP3 in Treg cells and the formation of gd-IgA1 by inhibiting the formation of Cosmc [15]. MiR-148b is not only a key miRNA responsible for the formation of gd-IgA1, but it is also likely involved in the injury of podocytes and renal tubules [9,58]. Both miR-320 and miR-374b are involved in the proliferation of B cells through PTEN and in the formation of gd-IgA1 by inhibiting Cosmc [14,16].

## 4. Association with the severity of renal damage

A large number of studies have reported that miRNAs are related to the severity of IgAN renal damage. However, few studies have determined that these miRNAs are specific to IgAN kidney damage compared with other forms of nephropathy.

The association between urine miRNA and the severity of IgAN has been most studied. Wang et al. [8] reported that the expression levels of miR-200a, miR-200b, and miR-429 in the urine of IgAN patients significantly and negatively correlated with proteinuria; miR-200b and miR-429 significantly positively correlated with baseline renal function; and miR-429 negatively correlated with the degree of glomerular scarring. Another study [60] indicated urinary miR-200b as a biomarker of renal fibrosis. In addition, it has been shown that the levels of miR-146a and miR-155 in the urine of IgAN patients were significantly increased and positively correlated with urine protein levels [50]. Another study reported [45] that the degree of proteinuria was significantly related to the urinary levels of miR-29b and miR-29c, while the estimated glomerular filtration rate(eGFR) was significantly related to the urinary levels of miR-21, miR-29b, and miR-29c. The level of miR-93 in urine was significantly related to glomerular scar formation. Szeto et al. [61] reported that miR-17 was up-regulated in IgAN and negatively correlated with baseline renal function. Wang et al. [62] compared the expression levels of miR-3613-3p and miR-4668-5p between IgAN, other diseases (membranous nephropathy and minimal change nephropathy), and healthy controls; the results showed that the urinary level of miR-3613-3p in IgAN was significantly lower than that in other nephropathies and healthy controls. The urinary level of miR-4668-5p in IgAN was also significantly lower than that in healthy controls. There was also a significant correlation between the urinary levels of miR-3613-3p and miR-4668-5p and 24-h urine protein excretion, eGFR and Lee classification, and the number of segmental sclerosis glomeruli in the Oxford classification. Szeto et al. [63] indicated that urinary miR-155 was significantly upregulated in the IgAN group and was associated with eGFR.

Renal miRNAs have been also explored in relation to the severity of IgAN. A study [50] has reported that the levels of intrarenal miR-146a and miR-155 are significantly higher in IgAN than in controls. The eGFR correlated negatively and proteinuria correlated positively with intrarenal levels of miR-146a and miR-155. Meanwhile, the intrarenal level of miR-155 significantly correlated with tubulointerstitial scarring. Other studies dealing with miR-146a and miR-155 [15,49] reported that the level of miR-155 in PBMCs of IgAN was significantly lower than that in healthy controls, and it was related to 24-h urine protein amount and urine red blood cell count. Moreover, in B6MRLc1 CKD mice (a mouse model of autoimmune glomerulonephritis), remarkably high levels of miR-146a are associated with severe interstitial lesions with cell infiltration, tubular atrophy, interstitial fibrosis, and glomerular lesions. Bao et al. [57] showed a significant increase in miR-21 in both glomerular and tubulointerstitial tissues of IgAN. The level of glomerular miR-21 positively correlated with the level of urine protein and glomerular sclerosis, while the level of tubulointerstitial miR-21 positively correlated with the level of urine protein, serum creatinine, and interstitial fibrosis. Osamu et al. [64] analyzed glomerular miRNA expression in B6.MRLc1 mice; found that the level of glomerular miR-26a in B6.MRLc1 was significantly lower than that in C57BL/6 (normal control) mice. In kidneys of B6.MRLc1 mice, podocytes mainly expressed miR-26a, and glomerular miR-26a expression negatively correlated with the urinary protein levels. However, in humans, the miR-26a levels in urinary exosomes were higher in patients with lupus nephritis (but not in patients with IgAN) than in healthy controls and correlated positively with urinary protein levels. Another study [65] revealed that renal expression levels of miR-21-5p, miR-214-3p, and miR-199a-5p were significantly associated with Oxford classification grade-T score (renal tubule atrophy or renal interstitial fibrosis). The expression level of miR-21-5p, but not miR-214-3p or miR-199a-5p, was associated with Oxford classification grade-S score (segmental sclerosis or adhesion). Lu et al. [58] revealed that renal miR-148b levels correlated positively with eGFR, but not with 24-h urinary protein excretion. Another study [66] revealed that the level of intrarenal miR-200c was downregulated, whereas the levels of intrarenal miR-141, miR-205, and miR-192 were upregulated in IgAN. Proteinuria significantly correlated with the intrarenal expression of miR-200c, and GFR significantly correlated with the intrarenal expression of miR-205. The degree of tubulointerstitial scarring correlated with miR-205 expression, whereas glomerulosclerosis correlated with miR-192 expression. However, in another study, miR-192 was lower in IgAN patients and it correlated with GFR [42]. Patients with lower intrarenal miR-192 levels had a higher degree of interstitial fibrosis, more severe lesions in tubular atrophy, and interstitial inflammation [42]. MiR-233 in glomerular endothelial cells (GEnCs) in IgAN patients correlated with urine protein level, urine erythrocyte count, blood creatinine level, glomerular endothelial proliferation score, and glomerular crescent rate [56].

Some miRNAs in blood have been linked to the severity of IgAN. Plasma miR-148a-3p level has been reported to positively correlate with eGFR [67]. Not only renal miR-192, but also serum miR-192 is related to eGFR, interstitial fibrosis, lesions in tubular atrophy, and interstitial inflammation [42]. MiR-374b level of B cells in peripheral blood positively correlates with urine protein level and pathological MEST score [14]. We summerized the role of miRNAs in severity of renal damage of IgAN in Table 1.

**Table 1.** microRNAs correlated with the severity of renal damage .

miRNA	Research	Published time	Impact factor	Patients		miRNA source	miRNA level	Indication
				IgAN	Control			
miR-200a	Wang G et al. [7]	2010	2.761	43	13	Urine	↓	Proteinuria
miR-200b	Wang G et al. [7]	2010	2.761	43	13	Urine	↓	Proteinuria Baseline GFR
miR-429	Wang G et al. [7]	2010	2.761	43	13	Urine	↓	Proteinuria Baseline GFR Degree of glomerular scar
miR-200C	Wang G et al. [65]	2009	3.684	43	13	Intrarenal	↓	Proteinuria
miR-205	Wang G et al. [65]	2009	3.684	43	13	Intrarenal	↑	Baseline GFR Degree of glomerular scar
miR-192	Wang G et al. [65]	2009	3.684	43	13	Intrarenal	↑	Glomerular sclerosis The rate of GFR decline
	Fan Q et al. [39]	2018	2.085	50	25	Intrarenal	↓	Baseline GFR interstitial inflammation and fibrosis tubular atrophy score
miR-146a	Wang G et al. [49]	2011	2.761	43	13	Urine intrarenal	↑	Proteinuria Baseline GFR
miR-155	Wang G et al. [49]	2011	2.761	43	13	Urine intrarenal	↑	Proteinuria Baseline GFR Renal tubulointerstitial scar
miR-21	Wang G et al. [45]	2012	2.761	43	13	Urine	↓	Baseline GFR
	BAO H [56]	2014	2.705	20	10	Intrarenal	↑	Proteinuria Glomerular sclerosis Serum creatinine Interstitial fibrosis
	Marie-Flore Hennino [31]	2016	4.011	56	–	Intrarenal	↑	T score in MEST score S score in MEST score
miR-29	Wang G et al. [45]	2012	2.761	43	13	Urine	↓	Proteinuria
miR-93	Wang G et al. [45]	2012	2.761	43	13	Urine	↑	Degree of glomerular scar
miR-17	Cheuk-Chun Szetoa [61]	2012	2.761	17	39^a^	Urine	↑	Baseline GFR
miR-223	Bao H [55]	2014	8.306	30	20	Intrarenal	↓	Proteinuria Urine RBC Serum creatinine glomerular EP score glomerular crescent rate
miR-26a	Osamu Ichii [64]	2014	2.776			Intrarenal	↓	Proteinuria
miR-374b	Yang B [76]	2015	2.675	30	15	Blood B cell	↑	Proteinuria MEST score
miR-3613-3p	Wang N [62]	2015	2.353	18	14^b^	Urine	↓	Proteinuria eGFR Lee’s grades
miR-4668-5p	Wang N [62]	2015	2.353	18	14^b^	Urine	↓	eGFR Lee’s grades
miR-214-3p	Marie-Flore Hennino [31]	2016	4.011	56		Intrarenal	↑	T score in MEST score
miR −199 -5p	Marie-Flore Hennino [31]	2016	4.011	56		Intrarenal	↑	T score in MEST score

^a^This study included 56 CKDs, the underlying histological diagnosis was IgA nephropathy (17 cases), diabetic nephrosclerosis (17 cases), and hypertensive nephrosclerosis (22 cases). There were no normal controls in this study. ^b^4 patients with MN (membranous nephropathy), 4 patients with MCD (minimal changes disease), and 6 healthy subjects. In addition, the rest of the control group were normal persons. GFR: glomerular filtration rate; T score: renal tubule atrophy or renal interstitial fibrosis; S score: segmental sclerosis or adhesion; glomerular EP score: glomerular endothelial proliferation score; Urine RBC: urine red blood cell.

## 5. Diagnostic potential

Currently, kidney biopsy is still the gold standard for diagnosing IgAN. Although accurate and specific, kidney biopsy is expensive and traumatic. Therefore, reliable and non-invasive diagnostic biomarkers are needed for IgAN. Many studies have explored miRNAs in blood as diagnostic markers, and promising results have been obtained. For example, Serino et al. [68] studied the combined diagnostic value of two classic miRNAs, serum miR-148b and let-7b, in IgAN. In the training cohort, the combination of these two miRNAs showed an extremely high diagnostic value, with an AUC of 0.82 and a cutoff value of 0.19 (sensitivity 76%, specificity 75%) in receiver operating characteristic (ROC) curve analysis. This result was verified in the verification cohort, with an AUC of 0.78. They also investigated whether the combination of these two miRNAs can distinguish IgAN from other types of primary glomerulonephritis. Based on their results, the combined miRNA analysis has a strong ability to distinguish IgAN patients from non-IgAN patients, with an AUC of 0.76 and a cutoff value of 0.19 (sensitivity 69%, specificity 76%) in ROC curve analysis. These results indicate that the combined miRNA analysis can be considered a good diagnostic biomarker with good specificity for IgAN. Another study found that plasma miRNAs, including four upregulated miRNAs (miR-148a-3p, miR-150-5p, miR-20a-5p, and miR-425-3p), had an AUC of 0.80 and 0.76 in ROC curve in the training and testing phases, respectively [67].

However, miRNAs in urine have a greater advantage than miRNAs in the blood because they are easy to collect and have very convenient detection technology. Duan et al. [69] reported that miR-25-3p, miR-144-3p, and miR-486-5p were significantly more expressed in IgAN (*n* = 93) than in the normal group (*n* = 82) or disease control (*n* = 40). These three miRNAs had good specificity and sensitivity for the diagnosis of IgAN, in which the AUC value of miR-486-5p was the largest at 0.935. The combination of the three miRNAs increased the AUC value to 0.940. Through CD235a magnetic bead sorting, it was also determined that these miRNAs mainly come from urethral red blood cells. Another study [70] showed that urine miR-34a, miR-205, miR-21, and miR-155 levels can distinguish IgAN patients from normal controls, with AUC values of 0.86, 0.85, 0.66, and 0.71, respectively. The cutoff values were as follows: miR-34a ≤ 0.047, miR-205 ≤ 0.209, miR-21 ≥ 0.461, and miR-155 ≤ 0.002. Another study [63] explored the diagnostic value of miR-150, miR-204, miR-431, and miR-55; the AUCs of these four ROC curves were statistically significant, and miR-204 had the highest AUC (0.976); however, the study did not further explore the diagnostic performance of combining miRNAs. For miR-204, a urinary level below 0.34 unit has a 100% specificity and 90.9% sensitivity in diagnosing IgAN, while the level above 1.70 unit has a 100% sensitivity and 55.5% specificity to exclude the diagnosis.

In fact, there are many miRNAs with diagnostic potential. For example, in patients with IgAN, the urine levels of miR-29b and miR-29c are significantly lower [45] while the urine levels of miR-93 are significantly higher than in healthy controls. The urine miR-3613-3p level in IgAN is significantly lower than that in membranous nephropathy, minimal change nephropathy, and healthy controls. The urine miR-4668-5p level in IgAN is also significantly lower than that in healthy people [62]. These data indicate that these urine miRNAs may have a good potential for diagnosis, and they should be fully developed in future studies.

## 6. Prediction of IgAN prognosis

The most promising use of miRNA levels in urine is to predict the prognosis of diseases. At present, there has been great progress in research related to the use of miRNAs to predict the prognosis of IgAN. However, there are still some problems: for example, small sample size; too short follow-up time; lack of Cox survival analysis; and lack of ‘hard end points’, which are usually determined as double Scr levels compared with a renal puncture, or ESRD or eGFR decreased by 50%. Wang et al. [8] showed that after 33.4 ± 12.6 months, the rate of renal function decline was significantly related to the urine expression of miR-200b and kidney miR-192. Szeto et al. [61] reported that in CKD patients, the rate of GFR decline positively correlated with the urine expression of miR-21 and miR-216a. However, these conclusions have not been verified in the IgAN subgroup. In another study [37], after 48.9 ± 38.1 months, using the best threshold determined by the maximum hazard ratio, both miR-21-5p and miR-214-3p were significantly associated with the risk of renal failure. Moreover, the renal survival rate of patients with miR-21-5p expression >2.06 or 214-3p expression >1.60 was significantly worse. Duan et al. [70] found that after 13.88 ± 6.00 months of follow-up of IgAN patients, miR-144-3p levels correlated positively with changes in eGFR and negatively with changes in 24-h urine protein. In addition, the level of miR-25-3p positively correlated with changes in eGFR. Of the 85 patients in the IgAN group, 38.82% (*n* = 33) achieved complete remission (CR) at the end of follow-up. However, there was no significant difference in urine sediment miRNA levels between the CR group and the non-CR group. Liang et al. [70] found no correlation between the eGFR loss rate and the baseline miRNA level after a follow-up of 15.19 months. The decrease in 24-h urine protein correlated positively with baseline miR-21 and negatively with miR-205. They also divided 51 patients into CR and non-CR groups. The baseline miR-205 level of the CR group was significantly higher than that of the non-CR group, while miR-21 and miR-146a levels were lower than those in the non-CR group. After grouping according to different treatment methods, it was reported that in patients treated with steroids and/or immunosuppressants, the baseline miR-146a level in the CR group was significantly lower than that in the non-CR group, while in patients treated with renin-angiotensin-system(RAS) blockers only, the baseline miR-205 level of the CR group was significantly higher than that of the non-CR group.

## 7. Future perspectives

In summary, there has been great progress in the research of miRNAs in IgAN since 2010, including their impact on the pathogenesis and progression of IgAN, their use as biomarkers to determine the severity of renal damage in IgAN, and their diagnostic and prognostic value. However, there are still some problems that need to be solved in future studies.

First, the pathogenesis and progression regulated by miRNA should be further illustrated. As for the pathogenesis, here, we summarized the majority of the articles published since 2010 and found that most of the studies focused on the mechanism by which miRNAs cause the production of gd-IgA1 and the imbalance of immune regulation. However, the excessive production of pathogenic IgA1, insufficient IgA clearance, and deposition of IgA in the mesangial region are also major pathogenesis factors of IgAN. For example, miR-133a, miR-133b, and miR-185 seem to be associated with IgA1 deposition, and miR-17-5p might be associated with mesangial proliferation and endocytic trafficking. However, these miRNAs have only been screened by high-throughput sequencing and are expressed at low levels in IgAN [32], so further studies are needed. In addition, IgAN is influenced by genetic factors, especially gene polymorphism, such as HLA, T cell receptor gene, renin-angiotensin system gene, uterine bead protein gene, or cytokine gene. Questions arise as to whether miRNAs are also regulated by these gene polymorphisms to affect IgAN, and whether the polymorphisms of miRNA-corresponding DNA are related to the incidence of IgAN. At present, a few studies in this field have been carried out. A single nucleotide polymorphism (rs2910164 C > G) in pre-miR-146a is associated with the expression of miR-146a. A study [71] revealed that rs2910164 did not correlate with the susceptibility to IgAN but the age of onset of IgAN in adult patients from a Chinese Han population. Another research group [71] revealed that rs2910164 may affect the susceptibility and severity of pediatric IgAN. Further studies are needed to validate these findings. In addition, the upstream and downstream molecules of miRNAs have to be discovered as well.

Second, the credibility and specificity of results should be improved. First, due to the small sample sizes as well as the lack of validation cohort and other glomerular disease controls, the current studies in IgAN should be further confirmed. In addition, because miRNAs in specific lymphocytes subgroups are difficult to analyze, PBMCs are currently used in the study of miRNAs. Future studies should isolate specific lymphocytes to minimize confounding factors. At the same time, studies on the effect of miRNA modulators and other RNA‑targeted agents need more development.

Third, strong analytical approaches should be developed to urinary miRNAs research. Urinary miRNAs have become a research hot spot because of noninvasive and convenient collection and analysis. Nevertheless, optimization and standardization of miRNA extraction and quantification from urinary sediment are challenging. Commonly used methods include ultracentrifugation, through the addition of macromolecular crowding reagents, such as polyethylene glycol, or paramagnetic particles. Although these methods can obtain more pure miRNAs, they require a larger amount of fluid and are difficult to carry out in clinical practice [72]. In addition, most of the studies on the role of miRNAs in the prognosis of IgAN had a short follow-up time, and they lacked survival analysis. The current judgment of prognosis lacks hard endpoints and depends on the decrease rate of eGFR and the decrease rate of urinary protein. Moreover, urinary miRNAs should be further studied in diagnosis and predicting patients’ survival in IgAN.

In addition, miRNA has great potential in targeted therapy. First, one miRNA can act on multiple targets through different pathways, thereby affecting multiple pathogenesis or progression mechanisms of IgAN; second, miRNAs are small, with known and conservative sequences, and they are easy to manufacture; third, the treatment method is simple, including local or gastrointestinal injection, and the tissue can fully absorb miRNAs [73]. However, the complex network relationships among miRNA and the human genome, transcriptome, and proteome in IgAN need further studies.

## 8. Conclusions

Overall, the role of miRNAs in IgAN has been more fully elucidated. The research has gradually shifted from the preliminary exploration stage to more detailed studies, including the identification of downstream targets of miRNAs, the precise location of miRNAs, and the study of combinations of miRNAs for improving the diagnostic potential. Urine miRNAs have become a focus of research due to their intrinsic advantages. However, the research of urinary miRNAs needs further improvement, especially in the field of diagnosis and prognosis prediction, which requires multicenter studies. The role of miRNAs in IgAN also provides a basis for their pharmacological research. More attention should be paid to the role of miRNA modulators and inhibitors in the treatment of IgAN in the future.

## Abbreviations

CIC: circulating immune complexes
DEGs: differentially expressed genes
ECM: extracellular matrix
eGFR: estimated glomerular filtration rate
EMT: epithelial-to-mesenchymal transition
ESRD: end-stage renal disease
gd-IgA1: galactose&hx00AD;deficient IgA1
glomerular EP score: glomerual endothelial proliferation score
HRMCs: human renal mesangial cells
IgAN: IgA nephropathy
IRAK1: IL-1 receptor-associated kinase 1
MCD: minimal changes disease
miRNA: microRNA
MN: membranous nephropathy
PBMCs: peripheral blood mononuclear cells
ROC: receiver operating characteristic
S score: segmental sclerosis or adhesion
TRAF6: TNF receptor associated factor 6
Treg cell: T regulatory cell
T score: renal tubule atrophy or renal interstitial fibrosis
Urine RBC: urine red blood cell
